# Intracellular invertase hyperproducing strain of *Saccharomyces cerevisiae* isolated from Abagboro palm wine

**DOI:** 10.1038/s41598-023-32289-x

**Published:** 2023-03-27

**Authors:** Oghenesivwe Osiebe, Isaac Olusanjo Adewale, Bridget Okiemute Omafuvbe

**Affiliations:** 1 Department of Biochemistry and Molecular Biology, Obafemi Awolowo University, Ile-Ife, Nigeria; 2 Department of Microbiology, Obafemi Awolowo University, Ile-Ife, Nigeria

**Keywords:** Biotechnology, Microbiology, Molecular biology

## Abstract

There is an ever-increasing demand for industrial enzyme, necessitating a constant search for its efficient producers. The isolation and characterization of invertase producer yeasts from natural palm wine is reported in this study. Yeasts were isolated from fresh palm wine obtained from Abagboro community Ile-Ife, Nigeria following standard methods. A total of six yeast strains were isolated from the palm wine. The strains were screened for their ability to produce invertase and the most efficient invertase producer was characterized and identified using phenotypic and molecular methods. Isolate C showed the highest invertase activity (34.15 µmole/ml/min), followed by isolate B (18.070 µmole/ml/min) and isolate A (14.385 µmole/ml/min). The identity of isolate C was confirmed by genotypic methods to be *Saccharomyces cerevisiae* (OL629078.1 accession number on NCBI database). The *Saccharomyces cerevisiae* strain fermented galactose, arabinose, maltose, glucose, sucrose and raffinose, grew in 50% and 60% glucose and at 25–35 °C. The newly isolated *Saccharomyces cerevisiae* strain is an efficient producer of invertase and can be exploited for commercial biosynthesis of the enzyme for use in biotechnological applications.

## Introduction

Palm wine, a naturally fermented alcoholic beverage is widely consumed in West Africa^1,2^. A vast microbiota is responsible for its attributes such as taste, ethanol content and aroma^3,4^. Various microorganisms of commercial importance have been isolated from the microbial pool present in palm wine^2,5^. The predominant microbes commonly isolated from palm wine microbiota community includes bacteria and fungi. The most common bacteria isolated include strains of the following family: *Acetobacteriaceae, Lactobacillaceae* and *Leuconostocaceae*, while the most frequently isolated yeast belong to the genera of *Saccharomyces cerevisiae* and *Candida parapsilosis*^2,5^. Strains of *Saccharomyces cerevisiae* are one of the most versatile cell factories applied for biotechnological applications including commercial enzyme production.

Enzymes are specific catalyzers and show high efficiency in acceleration of specific chemical reactions. In the range of a hundred million to ten billion times faster in speed of catalysis of reactions over the rates in the absence of enzymes^6^. As a result of this catalytic nature of enzymes, they find numerous applications in industrial and biomedical fields^7^. The industrial enzyme market around the globe was valued at USD 5.9 billion in 2020, and is expected to grow at a constant annual growth rate of 5.5% between 2020 and 2027 reaching about 8.7 billion by 2026^7,8^.

Invertase (β-fructofuranosidase) (E.C. 3.2.1.26) is one of such important industrial enzymes^9^. It finds applications in hydrolysis of sucrose containing substances, to yield a mixture of monosaccharide units (glucose and fructose) which have diverse applications, including the production of alcoholic beverages by fermentation. The production of non-crystallizable high fructose syrup is one of the most frequent use of invertase^10,11^. The enzyme is also important for the production of short chain fructooligosaccharides units and thus finds applications in pharmaceutical formulations of prebiotics^12^. Invertase is widely distributed in the biosphere, however *Aspergillus niger* and *Saccharomyces cerevisiae* are the major cell systems used in commercial synthesis of invertase^9^. *Saccharomyces cerevisiae* produces both intracellular and extracellular forms of invertase. However, much investigation on isolation and identification of efficient invertase producers have been focused solely on extracellular invertase, creating a dearth of information.

There is an ever-increasing demand for industrial enzymes and a constant search for efficient producers. As the result of certain advantages such as: not complicated production and product recovery, economic feasibility, consistent yields, constant availability, ease of genetic manipulations and ethical issues, microorganisms are preferred over other sources of enzymes^6,13^. The aim of this study was to isolate and characterize (phenotypically and molecularly) intracellular invertase hyperproducing yeasts from palm wine collected from Abagboro community Ile-Ife, Nigeria.

## Materials and methods

### Sample collection and isolation procedure

Freshly tapped palm wine was collected from Abagboro community, Ile-Ife, Osun state, Nigeria. Collected palm wine was immediately transported to the laboratory in ice bucket for analysis. The palm wine was serially diluted in sterile maximum recovery diluent (MRD, Oxoid) and 0.1 ml of appropriately diluted palm wine was inoculated onto Sabouraud dextrose agar (SDA) plates containing 0.01% chloramphenicol (to exclude the growth of bacteria) using spread plate method^14^. The SDA plates were incubated at 28 °C for 48 h. After incubation period, the colonies were selected based on morphology and representative colonies were purified by repeated streaking on SDA followed by incubation under similar conditions. Pure isolates were transferred to SDA slants, assigned isolate codes (A–F) and maintained in the refrigerator at 4 °C and sub-cultured periodically until they were screened to select the highest producer of invertase and identified.

### Screening for efficient intracellular invertase producing isolate

A loop full of pure yeast isolates from each representative colony was inoculated into sterile broth medium^15^. Sterile broth medium containing sucrose (2%) as the only carbon source, yeast extract (0.4%), potassium dihydrogen phosphate (KH_2_PO_4_) (0.1%) and magnesium sulfate heptahydrate (MgSO_4_.7H_2_0) (0.05%)^16^. Yeast was incubated for 72 h at 28 °C in the media after which cells were harvested by centrifugation at 10,000 × g at 4 °C for 30 min. Harvested cells were homogenized with acid-washed sea sand in Tris–HCl buffer pH 7.5, ratio 1:1:2 respectively, using a mortar and pestle^17^. Homogenate was centrifuged at 10,000 × g at 4 °C for 30 min to obtain supernatant. The activity of invertase in the supernatant was quantified by enzyme assay^18^.

### Enzyme assay

The amount of invertase present in the supernatant was quantified by measuring the quantity of reducing sugar released when the enzyme was incubated for 10 min with 0.143 mM sucrose (substrate) in 80 mM sodium acetate buffer pH 4.7 at 28 °C following the Somogyi- Nelson method^18^. The blank control contained boiled enzyme solution. One unit of enzyme activity was defined as the quantity of the enzyme releasing reducing sugars equivalent to 1 μmole of glucose from sucrose per minute at room temperature.

### Characterization of selected invertase producing yeast

The selected high-invertase producing yeast was characterized using phenotypic method and the ribosomal Internal Transcribed Spacer (ITS) region gene sequence analysis. Phenotypically, the isolate was examined microscopically using lactophenol cotton blue test according to the method of Fawole and Oso^19^. Sporulation test (ascospore formation) was done according to the method of Kurzman et al.^10^ using Gorodokwa agar (sporulation medium) and malachite green staining technique to detect ascospore. Sugar fermentation was done following the method of Kurzman et al.^20^. A light suspension of yeast cells was prepared in a test tube by suspending 18–24 h old cell cultures in about 5 ml of distilled water. This was viewed against a black line of about 3/4 mm wide until it was visible as dark bands. The light suspension prepared was inoculated (0.1 ml) into the fermentation medium (yeast extract, peptone water, sugar (fermentable substrate), bromocresol purple (indicator) with inverted Durham tubes in a test tube). Sugar substrate tested included 2% of various sugars such as glucose, mannitol, maltose, arabinose, sucrose, lactose, galactose and 4% of raffinose. The inoculated media containing various sugars was incubated at 30 °C for 14 days and then observed for colour change and production of gas. A change in the indicator colour from purple to yellow indicated a positive result, while downward displacement of the fermentation medium in the Durham tube indicated a high fermentative rate. Sugar tolerance test was performed by growing the isolate in broth medium containing 1% yeast extract and either 50% or 60% glucose^20^. The medium (10 ml) was inoculated with 0.1 ml each of light yeast cell suspension (prepared from 18 to 24 h old cell culture) and incubated at 30 °C for 72 h^10^. During this period, the growth rate of the isolate was monitored at 600 nm^21^. Temperature tolerance of the isolate was tested in sterile broth medium (consisting glucose, peptone and yeast extract). The medium (10 ml) was inoculated with 0.1 ml of light yeast cell suspension (prepared from 18 to 24 h old cell culture) and incubated at temperatures ranging from 30 to 40 °C for 72 h and observed for growth^20^.

### Molecular characterization of selected invertase producing yeast

The isolate genomic DNA was extracted as previously described^22^. The most efficient invertase producer was identified by ribosomal Internal Transcribed Spacer (ITS) region sequencing using forward primer (ITS1) 5′-TCCGTAGGTGAA CCTGCGG-3′ and reverse primer (ITS4) 5′-TCCTCCGCTTATTGATATGS-3′ following the method of by Sebastiani et al.^23^. Amplified sequence was verified by viewing on 1.5% Agarose electrophoresis, purified and sequenced with Applied Biosystems 3500 Genetic Analyzer and AB1 sequence obtained were exported to FASTA format and submitted to GenBank. Basic Local Alignment Search Tool (BLAST) on the National Centre for Biotechnology Information (NCBI) database was used to find sequence with significant similarity and to confirm the specie type.

### Phylogenetic analysis

Ribosomal Internal Transcribed Spacer (ITS) that had significant sequence similarity with the sequence ITS in this study were multiple-aligned using Clustal W algorithm. Phylogenetic tree generated in this study was constructed using a neighbor-joining algorithm, where a pairwise distance matrix for all the sequences to be aligned was first generated, subsequently a guide tree was created for phylogeny. Clustal Omega (https://www.ebi.ac.uk/Tools/msa/clustalo/) online platform was used for all phylogeny processes. The guide tree format result was exported and uploaded to the Interactive Tree of Life (iTOL) version 5 (https://itol.embl.de), which is an online tool for displaying and annotating phylogenetic trees.

## Results

### Isolation and screening of high invertase-producing yeast from Abagboro palm wine

**Figure 1:**
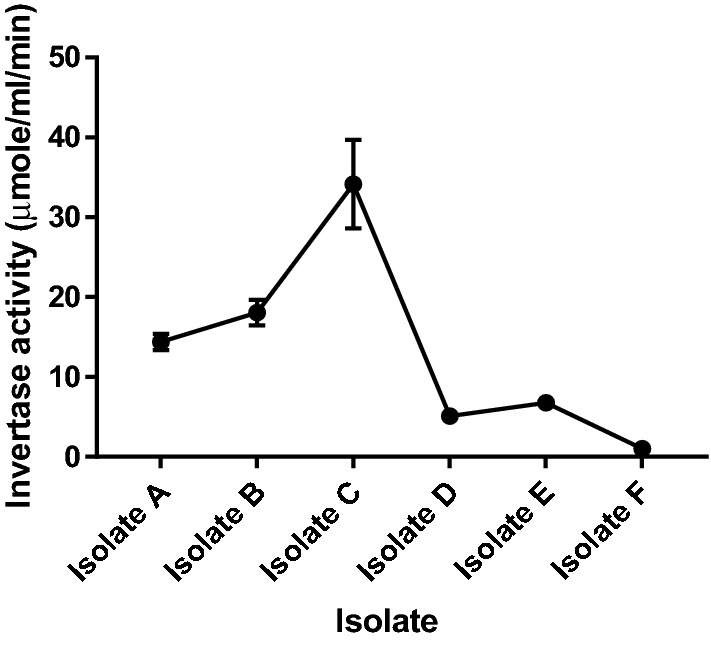
Invertase activity of six yeast isolates obtained from Abagboro palm wine. Values are mean of three determinations; bars represent standard error of mean.

Six yeast isolates were recovered from palm-wine obtained from Abagboro community, Ile-Ife, Nigeria. The isolates showed different levels of expression of intracellular invertase as shown in Fig. 1. All the yeast isolates examined expressed intracellular invertase in different quantities, in terms of enzyme units present (1.005–38.640 µmole/ml/min). Isolates A, B and C showed the highest invertase activity among the strains isolated and were biochemically and morphologically characterized. The highest invertase producer (isolate C) was subsequently confirmed using molecular method.

**Figure 2:**
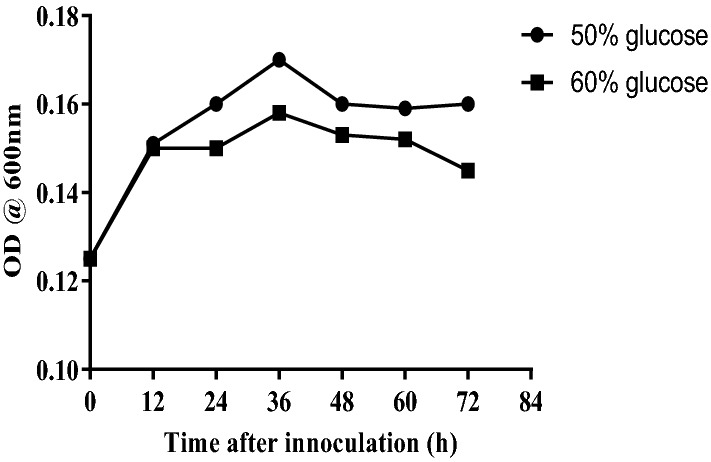
Growth of isolate C (*Saccharomyces cerevisiae*) in medium containing 50% and 60% glucose.

The morphological properties of isolates A, B and C after culture on Sabouraud dextrose agar plate at 30 °C for 18 to 24 h is presented in Table 1. Microscopically, the isolates were similar; ovoid to circular in shape, with size ranging from 2 to 6 μm, and was observed to have budding cells. The isolates had morphological characteristics which correspond to *Saccharomyces* type unicellular ascomycete. Isolate C utilized galactose, arabinose, maltose, sucrose and raffinose in addition to glucose, based on the sugars fermented, it was classified as *Saccharomyces cerevisiae,* based on fermented sugars isolate A and B were classified as *Saccharomyces paradoxus* (Table 2)*.* Isolate A and B did not grow in 50% and 60% glucose. Isolate C showed good growth rate when it was cultured in medium supplemented with 50% and 60% glucose (Fig. 2) and temperature of 35 °C (Table 2).

**Table 1:** Morphological characterization of Palm wine yeast.

Character	Isolate code		
	A	B	C
Type of edge	Entire	Entire	Entire
Size	Small	Medium	Medium
Shape	Circular	Circular	Circular
Elevation	Raised	Raised	Raised
Opacity	Opaque	Opaque	Opaque
Colour	White	White	White
Surface	Smooth	Smooth	Smooth
Sporulation test	+	+	+

Key:

+, Positive.

**Table 2:** Biochemical and physiological characterization of Palm wine yeast.

Isolate code	Sugar fermentation test								Sugar tolerance test		Thermal tolerance test				Probable identity
	Mannitol	Maltose	Raffinose	Galactose	Arabinose	Glucose	Lactose	Sucrose	Growth in 50% glucose solution	Growth in 60% glucose solution	Growth at 25 °C	Growth at 30 °C	Growth at 35 °C	Growth at 40 °C	
A	−	−	+	−	−	+	−	+	−	−	+	+	+	−	*Saccharomyces paradoxus*
B	−	−	+	+	−	+	−	+	−	−	+	+	+	−	*Saccharomyces paradoxus*
C	−	+	+	+	+	+	−	+	+	+	+	+	+	−	*Saccharomyces cerevisiae*

Key:

−,  Negative.

+,  Positive.

### Molecular characterization of isolate C

**Figure 3:**
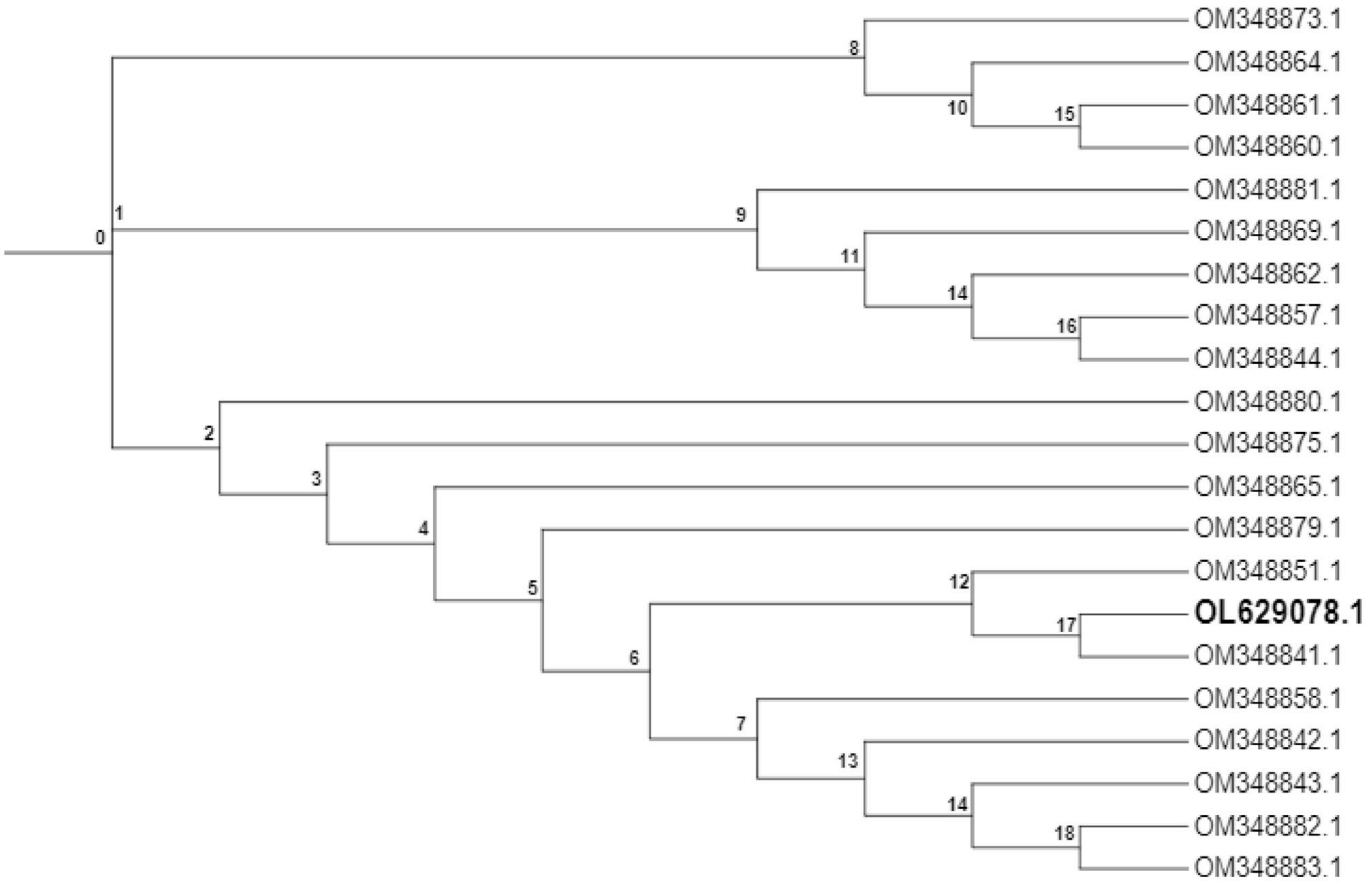
The phylogenetic tree of *Saccharomyces cerevisiae* isolate OL629078.1. Isolate in this study clustered with the strain OM348841.1 at clade 17, hereby having significant strain similarity, diverging from other strains isolates.

The identity of the efficient invertase producing yeast isolate was confirmed by ribosomal Internal Transcribed Spacer (ITS) region (rDNA ITS) as *Saccharomyces cerevisiae* and the sequence was deposited on the GenBank and the accession number OL629078.1 was attributed to the isolate*.* Phylogeny of the isolate C is shown in Fig. 3. The *Saccharomyces cerevisiae* in this study with accession number; OL629078.1 (shown in bold) has significant sequence similarity with *Saccharomyces cerevisiae* strain OM348841.1, shown in the phylogenetic tree (Fig. 3) as they clustered at clade 17, diverging from other strains.

## Discussion

Palm wine is the alcoholic drink that results from spontaneous natural fermentation of palm tree sap, making it a natural store for microorganisms potentially useful in biotechnology applications. Various strains of important microbial cell factories, commonly employed in the production of microbial enzymes such as *Saccharomyces* spp. and *candida* spp. have been frequently isolated from the rich microorganism store of palm wine^2,5,7^. Yeast isolates in this study showed varied levels of expression of intracellular invertase indicating the biodiversity of the microbial community present in the palm wine. Isolate C proved to be a natural invertase hyperproducing strain of *Saccharomyces cerevisiae* (34.15 U/ml). Similar range of invertase production (34.1 U/ml) have been observed for *Aspergillus niger* extracellular invertase hyperproducing strain^24^. Oyedeji et al.^11^ reported a maximum extracellular invertase yield of 24 U/ml for *Aspergillus niger* grown on pineapple peels. Ali and Ul-haq^25^ reported a maximum yield of 21.48 U/min (107 U/5 min) of extracellular invertase for *Saccharomyces cerevisiae* isolated from dates. Alegre et al.^26^ investigated the effect of various carbon sources on invertase production and reported a maximum yield of 15 U/ml for intracellular invertase production by *Aspergillus caespitosus.* Isolate C was identified and phenotypically characterized as unicellular ascomycete of the *Saccharomyces* type.

The ability of yeast to ferment various sugars other than glucose is a property which is important for classification of yeast and in assessing potential strains of interest in industries. The selected yeast was able to ferment arabinose, galactose, sucrose and raffinose in addition to glucose. The fermentation of arabinose is a unique feature of the isolate when compared to *Saccharomyces cerevisiae* from other sources^27,28^. Cell factories which are capable of converting all sources of sugar in feedstock into valuable end products are in constant demand in biotechnological processes. Sustainable bioethanol production is dependent on the availability of sturdy ethanol producers which is able to metabolize different carbon sources present in feedstock for the production of biofuel. Therefore, the ability to grow in arabinose is a potential which can be harnessed for improved biotechnological applications.

In addition to fermentation of diverse sugars, sugar tolerance is an important property when assessing the suitability of isolates for various applications including the synthesis of functional foods and alcoholic beverages. The isolated yeast showed good growth rate when it was cultured in medium containing 50% and 60% glucose, an indication of its ability to withstand osmotic stress, a potential which can be harnessed in processes involving high glucose solutions. Since osmophillic yeast preparations have been known to produce polyalcohols, we allude that the isolate would be particularly suitable for such applications. In addition, various studies have reported a direct relationship between sugar-tolerance and ethanol-tolerance^29,30^, suggesting that the yeast would be suitable in alcoholic fermentations for the production of alcohols. The yeast showed good growth rate between 25 and 35 °C, suggesting its suitability for use in bioreactors. The utilization of thermotolerant organisms for fermentation is desired due to an increase in the rate of reactions related to fermentation, reduction in cooling cost and reduction of contamination by mesophilic organisms. The phylogenetics of the ITS region (ascension number OL629078.1) shows variations with other *Saccharomyces cerevisiae* ITS, but shared same clade with *Saccharomyces cerevisiae* strain OM348841.1.

## Conclusion

In conclusion, the newly identified yeast (*Saccharomyces cerevisiae*) isolated from Abagboro palm wine is an efficient producer of intracellular invertase which has suitable physiological properties for biotechnological applications. The study revealed that palm wine obtained from Abagboro community, Ile-Ife, Osun state could be a source of *Saccharomyces cerevisiae* strains for various biotechnological applications.

## Data Availability

Data analyzed in the current study is available at GenBank National Centre for Biotechnology Information database [https://www.ncbi.nlm.nih.gov/nuccore/OL629078.1].
